# A Commonly Used Biocide 2-N-octyl-4-isothiazolin-3-oneInduces Blood–Brain Barrier Dysfunction via Cellular Thiol Modification and Mitochondrial Damage

**DOI:** 10.3390/ijms22052563

**Published:** 2021-03-04

**Authors:** Donghyun Kim, Eun-Hye Kim, Sungbin Choi, Kyung-Min Lim, Lu Tie, Arshad Majid, Ok-Nam Bae

**Affiliations:** 1 College of Pharmacy Institute of Pharmaceutical Science and Technology, Hanyang University, Ansan 15588, Korea; ssks7787@naver.com (D.K.); rladmsgp615@naver.com (E.-H.K.); hjklkl123@naver.com (S.C.); 2 College of Pharmacy, Ewha Womans University, Seoul 03760, Korea; kmlim@ewha.ac.kr; 3 Department of Pharmacology, School of Basic Medical Science, Peking University, Beijing 100083, China; tielu@bjmu.edu.cn; 4 Sheffield Institute for Translational Neuroscience, University of Sheffield, Sheffield S10 2TN, UK; arshad.majid@sheffield.ac.uk

**Keywords:** isothiazolinone (IT) biocide, 2-n-Octyl-4-isothiazolin-3-one (OIT), blood–brain barrier (BBB) model, protein S-nitrosylation (SNO), mitochondrial dysfunction, oxidative stress

## Abstract

Isothiazolinone (IT) biocides are potent antibacterial substances commonly used as preservatives or disinfectants, and 2-n-Octyl-4-isothiazolin-3-one (OIT; octhilinone) is a common IT biocide that is present in leather products, glue, paints, and cleaning products. Although humans are exposed to OIT through personal and industrial use, the potentially deleterious effects of OIT on human health are still unknown. To investigate the effects of OIT on the vascular system, which is continuously exposed to xenobiotics through systemic circulation, we treated brain endothelial cells with OIT. OIT treatment significantly activated caspase-3-mediated apoptosis and reduced the bioenergetic function of mitochondria in a bEnd.3 cell-based in vitro blood–brain barrier (BBB) model. Interestingly, OIT significantly altered the thiol redox status, as evidenced by reduced glutathione levels and protein S-nitrosylation. The endothelial barrier function of bEnd.3 cells was significantly impaired by OIT treatment. OIT affected mitochondrial dynamics through mitophagy and altered mitochondrial morphology in bEnd.3 cells. N-acetyl cysteine significantly reversed the effects of OIT on the metabolic capacity and endothelial function of bEnd.3 cells. Taken together, we demonstrated that the alteration of the thiol redox status and mitochondrial damage contributed to OIT-induced BBB dysfunction, and we hope that our findings will improve our understanding of the potential hazardous health effects of IT biocides.

## 1. Introduction

Isothiazolinone (IT) biocides are antibacterial substances commonly used as preservatives or disinfectants in commercial products [1,2]. Although the use of antibacterial substances is increasing globally, the systemic effects of IT biocides on human health are poorly understood [3,4,5,6]. These biocides can be absorbed orally and dermally during personal and industrial use [7,8,9] and subsequently circulate in the blood posing a human health risk [2,10,11]. While blood vessels are continuously exposed to circulating xenobiotics, the effects of IT biocides on the vascular system remain unknown. Among several forms of IT biocides, 2-n-octyl-4-isothiazolin-3-one (OIT; octhilinone) is commonly used in leather and textile products as a preservative. OIT shows high skin-penetration potency [12], suggesting its potential for systemic circulation following dermal exposure. Several health effects of occupational or non-occupational OIT exposure have been reported in humans [12,13,14,15]. The hetero-aromatic ring structure of OIT is involved in the mechanisms of biocidal effects, because it has strong reactivity toward the cysteine thiol residues in living organisms [16]. Several studies have suggested that glutathione (GSH) depletion might be the main toxicity mechanism of IT biocides, as observed in human leukemia cells [17,18].

The blood–brain barrier (BBB) is a vascular endothelial cell (EC) system in the brain, tightly limiting the transportation of molecules from the blood to the central nervous system [19,20,21]. The BBB constitutes the neurovascular unit, which emphasizes the structural and functional relationship between neuronal, glial, and vascular cells, and plays a major role in regulating the brain microvascular environment [19]. Environmental pollutants can contribute to cerebrovascular diseases such as ischemic stroke [22,23], and many studies have reported that xenobiotics may initiate BBB impairment and lead to central nervous system damage [22]. In this context, the BBB is a vascular target for xenobiotics circulating in the blood as well as an important determinant of neuronal damage. Notably, brain ECs show a high demand for ATP to maintain their barrier function, and mitochondrial damage can contribute to the dysfunction of brain ECs [24,25,26]. Accumulating evidence has shown that mitochondrial dysfunction and the formation of mitochondria-associated reactive oxygen species (ROS) are one of the major molecular mechanisms of endothelial dysfunction [27,28]. The mitochondrial contents in brain ECs are relatively higher than those in other organs [29]. During mitochondrial quality control, damaged mitochondria are removed by mitochondria-specific autophagy, also called mitophagy [30,31]. The removal and biogenesis of mitochondria are tightly regulated, because excessive removal of mitochondria can reduce the number of healthy mitochondria involved in energy production and cell signaling that is required to maintain normal physiology [30]. We have recently shown that excessive activation of mitophagy is related to pathological conditions in ECs [21].

S-nitrosylation (SNO) is a post-translational modification involving the reversible reaction of nitric oxide (NO) with protein thiol groups (sulfhydryls) of cysteine residues [32]. In ECs, the half-life of SNO is approximately 1 h, and S-nitrosoprotein formation increases with the endogenous production of NO by endothelial NO synthase [33]. A recent study suggested that S-nitrosoproteins also exist in the mitochondria and that SNO might change the activity of the electron transport chain (ETC) [33,34,35]. However, the exact role of SNO during the pathological process of brain ECs needs further investigation.

In this study, we aimed to examine the effects of OIT on BBB function and elucidate its underlying mechanisms, especially regarding thiol modification and the alteration of mitochondrial function.

## 2. Results

### 2.1. Impact of OIT on bEnd.3 Cell Deaths

**Figure 1:**
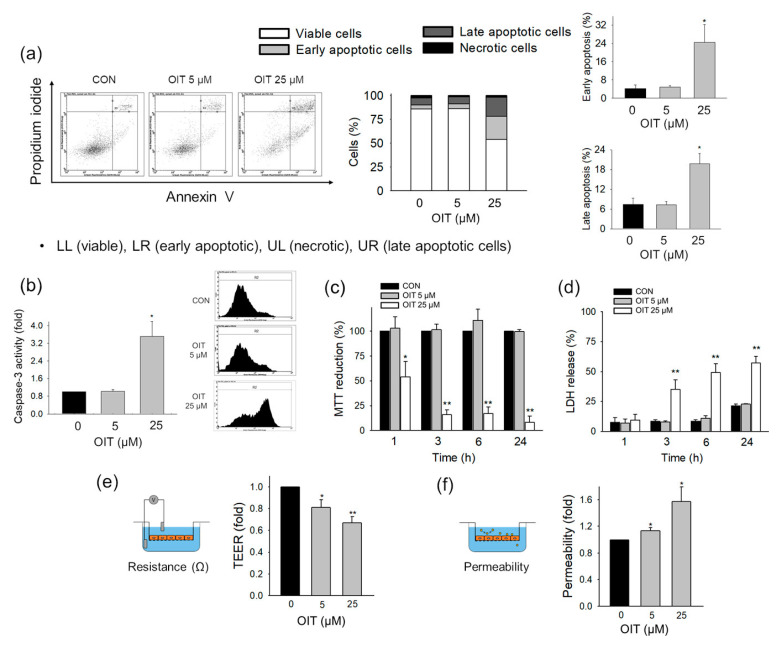
Effects of 2-n-Octyl-4-isothiazolin-3-one (OIT) on the apoptosis pathway, metabolic capacity, plasma membrane damage, and endothelial function in bEnd.3 cells. (**a**) Early-apoptotic, late-apoptotic, and necrotic cells were analyzed at 24 h after treatment with 0, 5, or 25 μM OIT (*n* = 4). (**b**) Caspase-3 activity was examined at 3 h after treatment with 0, 5, or 25 μM OIT (*n* = 4). (**c**,**d**) The extents of (**c**) MTT reduction and (**d**) LDH release were examined at 0, 1, 3, 6, and 24 h after treatment with 0, 5, or 25 μM OIT (*n* = 3–6). (**e**,**f**) Functional changes in endothelial permeability were analyzed by TEER measurements (**e**) and an in vitro FITC-dextran (m.w. 4000 Da) permeability assay (**f**) at 24 h after treatment of OIT (*n* = 3–4). Data are presented as mean ± SEM. * *p* < 0.05, ** *p* < 0.01 vs. CON (control).

First, we investigated the effects of OIT, an IT biocide, on cell death using brain endothelial bEnd.3 cells as in vitro BBB models. We examined the OIT-induced phosphatidylserine (PS) exposure and plasma membrane leakage, which are phenomena observed in apoptosis and necrosis [36]. Both early and late apoptotic cells significantly increased 24 h after treatment with 25 μM OIT (Figure 1a). Since caspase-3 plays a pivotal role during the intrinsic or extrinsic pathway of apoptosis [37], we examined caspase-3 activity after OIT treatment. Caspase-3 activity significantly increased 3 h after treatment with 25 μM OIT (Figure 1b). To identify the time course of OIT-induced changes in metabolic capacity and plasma membrane integrity, we examined the MTT reduction and LDH release 1, 3, and 6 h after OIT treatment. MTT reduction was significantly impaired by OIT 1 h after treatment, while the release of LDH increased 3 h after treatment (Figure 1c,d).

### 2.2. Impairment of Endothelial Barrier Function of bEnd.3 Cells by OIT

Next, we examined whether the barrier function of the in vitro BBB was altered by OIT exposure. The barrier function of the BBB is critical in maintaining homeostasis in the central nervous system (CNS) [38], and dysfunction of the BBB and increased permeability are associated with various neurological diseases. To investigate the effects of OIT on in vitro BBB function, we examined trans-endothelial electrical resistance (TEER) and endothelial permeability of FITC-dextran in bEnd.3 cells after OIT treatment. TEER significantly decreased after treatment with 5 and 25 μM OIT (Figure 1e). Consistent with this result, FITC-dextran permeability significantly increased after treatment with 5 and 25 μM OIT (Figure 1f). These observations suggest that functional impairment was induced by OIT treatment at a low concentration of 5 μM, the concentration that maintained the cell viability.

### 2.3. Tight Junction Protein Degradation and Modification of Intracellular Thiol Status Induced by OIT

**Figure 2:**
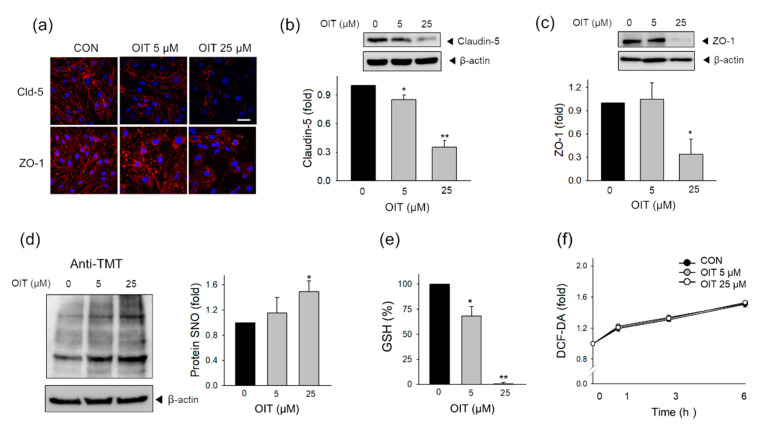
Effects of OIT on tight junction protein expression, protein S-nitrosylation (SNO), reduced glutathione (GSH), and total cellular ROS levels. (**a**) Immunofluorescence staining with claudin-5 or ZO-1 was performed in the OIT-exposed bEnd.3 cells and visualized by confocal microscopy at 24 h after OIT treatment (*n* = 3). Scale bar: 20 μm. (**b**,**c**) The protein levels of (**b**) claudin-5 and (**c**) ZO-1 were determined 24 h after treatment with 5 and 25 μM OIT (*n* = 3). (**d**) The level of SNO-modified proteins was detected at 1 h after treatment with 0, 5, or 25 μM OIT by the detection of the TMT-labeled S-nitrosylated proteins in western blot (*n* = 3). (**e**) The level of reduced GSH was measured at 24 h after treatment with OIT (*n* = 3). (**f**) Total cellular ROS was detected at 0, 1, 3, and 6 h after treatment with OIT (*n* = 3). Data are presented as mean ± SEM. * *p* < 0.05, ** *p* < 0.01 vs. CON (control).

As tight junction (TJ) proteins play a major role in maintaining endothelial function [39], we investigated whether OIT-induced functional impairment was related to changes in TJ proteins. First, to examine TJ protein expression and disposition, we visualized the tight junction proteins, including zonula occludens-1 (ZO-1) and claudin-5, using a confocal microscope. The immunofluorescence of claudin-5 and ZO-1 decreased after OIT treatment (Figure 2a). These results were well correlated with the results of Western blot analysis where the protein levels of claudin-5 significantly decreased by 0.85-fold and 0.35-fold, respectively, after treatment with 5 and 25 μM OIT compared with those in the control group (Figure 2b). The protein levels of ZO-1 significantly decreased by 0.30-fold after treatment with 25 μM OIT compared with those in the control group (Figure 2c).

Growing evidence has shown that the protein SNO is related to alterations in barrier function and metabolic capacity of endothelial cells [34,40,41,42]. As IT derivatives show high reactivity with thiols [16], we investigated the effects of OIT on the level of SNO-modified proteins in whole lysates by detecting TMT-labeled proteins. The levels of SNO-modified protein significantly increased 1 h after treatment with 25 μM OIT (Figure 2d). Since GSH is an intracellular antioxidant involved in the defense against oxidative stress, oxidation-reduction reactions in several metabolic pathways, and redox signaling [43], we subsequently measured the effect of OIT on the intracellular levels of reduced GSH. OIT significantly decreased the levels of reduced GSH in a dose-dependent manner (Figure 2e). However, the total cellular level of reactive oxygen species did not increase in bEnd.3 cells until 6 h after OIT treatment (Figure 2f), suggesting that this might not be the key mechanism of OIT effects in brain ECs.

### 2.4. OIT-Induced Mitochondrial Defects

**Figure 3:**
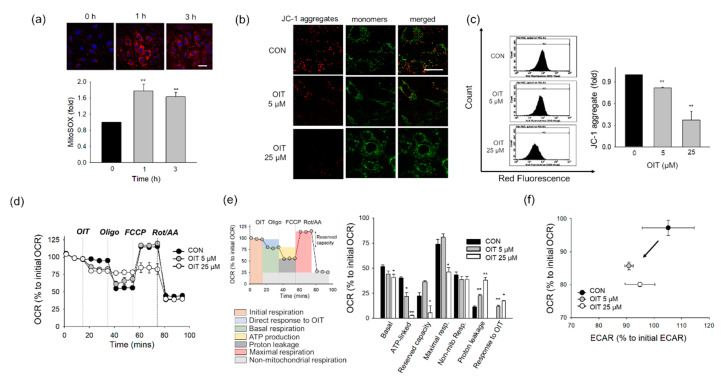
Changes in mitochondrial membrane potential and bioenergetics after OIT treatment in bEnd.3 cells. (**a**) Mitochondrial ROS was measured 1 and 3 h after treatment with 25 μM OIT (*n* = 3–6). Scale bar: 20 μm. (**b**) JC-1 aggregates (red) and monomers (green) were detected 3 h after OIT treatment by confocal microscopy. Scale bar: 20 μm. (**c**) Fluorescence intensity of JC-1 aggregates was measured 3 h after OIT treatment by a flow cytometer (*n* = 3). (**d**–**f**) bEnd.3 cells were subjected to Seahorse MitoStress Assay with an acute injection of OIT (*n* = 3). (**d**) The profile of the oxygen consumption rate (OCR) was plotted. (**e**) Parameters of mitochondrial respiration were calculated (**f**) Extracellular acidification rate (glycolysis) and oxidative phosphorylation (OCR) values were plotted. Arrows indicate metabolic changes between control (vehicle) and OIT-treated cells. Data are presented as mean ± SEM. * *p* < 0.05, ** *p* < 0.01 vs. CON (control).

Mitochondria are organelles engaged in bioenergetics and stress sensing for cellular adaptation to the environment [44]. Furthermore, mitochondria are the main ROS source, and mitochondrial ROS are generated after leakage from the ETC [31,45]. To investigate the effects of OIT on mitochondrial ETC, especially in proton leakage, we detected mitochondrial ROS using the MitoSOX Red mitochondrial superoxide indicator. The mitochondrial ROS level significantly increased 1 and 3 h after treatment with 25 μM OIT(Figure 3a).

As metabolic changes and mitochondrial ROS might be indicators for mitochondrial damage, we measured the mitochondrial membrane potential (MMP) and bioenergetic function of mitochondrial ETC after OIT treatment. First, we examined MMP using the JC-1 dye, which emits red fluorescence as aggregates in healthy cells with high ΔψM, but green fluorescence as monomers in cells with low ΔψM. The red fluorescence of JC-1 aggregates significantly decreased, while the green fluorescence of JC-1 monomers increased 3 h after OIT treatment in confocal microscopic observation (Figure 3b). Consistently, the amount of JC-1 aggregates significantly decreased 3 h after treatment with 5 and 25 μM OIT, as measured using flow cytometry (Figure 3c).

Next, we investigated the effects of OIT on bioenergetic oxidative phosphorylation in bEnd.3 cells. The oxygen consumption rate (OCR) decreased immediately after the addition of OIT to cells in a dose-dependent manner (Figure 3d,e). The bioenergetic parameters such as ATP-linked OCR and maximal respiration significantly decreased, and proton leakage significantly increased after OIT treatment. The ratio between OCR and ECAR was used to examine bioenergetics in cells, including oxidative phosphorylation and glycolytic potential (Robinson et al., 2012). After OIT treatment, both OCR and ECAR decreased, indicating that the cells showed low metabolic activity (Figure 3f).

### 2.5. OIT-Induced Mitophagy and Changes in Mitochondrial Mass and Morphology

**Figure 4:**
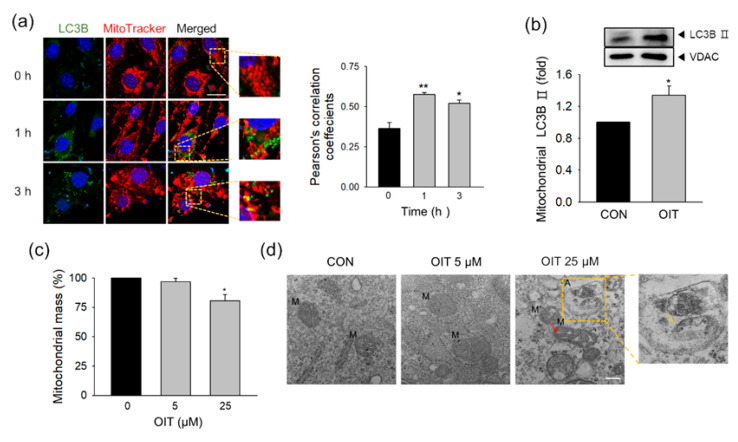
OIT-induced changes in mitophagy, mitochondrial mass, and mitochondrial morphology in bEND.3 cells. (**a**) The localization of LC3B (autophagosome) and MitoTracker (mitochondria) was examined 0, 1, and 3 h after treatment with 25 μM OIT by confocal microscopy. Scale bar: 20 μm. Pearson’s correlation coefficient was calculated from three independent experiments using ImageJ software (*n* = 3). (**b**) LC3B-II protein levels were detected in the mitochondrial fraction 3 h after treatment with 25 μM OIT (*n* = 3). (**c**) Mitochondrial mass was examined 3 h after treatment with OIT by nonyl acridine orange (NAO) staining (*n* = 3). (**d**) Changes in mitochondrial morphology and autophagosome formation were detected 3 h after treatment with 5 and 25 μM OIT by TEM (*n* = 3). M, mitochondria; A, autophagosome; red arrow, swollen mitochondria with distorted cristae; yellow box and yellow arrow, mitochondria-contained vesicle. Scale bar: 200 nm. Data are presented as mean ± SEM. * *p* < 0.05, ** *p* < 0.01 vs. CON (control).

Since mitochondrial quality control systems are crucial for maintaining mitochondrial function [46], we investigated the effect of OIT on mitochondria-specific autophagy, mitophagy, which is involved in the specific removal of impaired mitochondria. Confocal microscopy revealed that Light Chain 3B (LC3B), an autophagic indicator, was co-localized with the MitoTracker, a red-fluorescent dye that stains mitochondria, after OIT treatment (Figure 4a). The level of LC3B-II, a marker of autophagosome formation, significantly increased in the mitochondrial fraction (Figure 4b). The activation of mitophagy is known to affect mitochondrial dynamics and change the mass of mitochondria. The mitochondrial mass in brain ECs significantly decreased 3 h after treatment with 25 μM OIT (Figure 4c). Next, we examined the OIT-induced changes in mitochondrial morphology and autophagosome formation using transmission electron microscopy (TEM). Swollen mitochondria with distorted cristae were observed, and mitochondria-containing vesicles, which represent the stress-induced mitophagy in organs with high ATP demands, were found in OIT-treated bEnd.3 cells (Figure 4d).

### 2.6. NAC Effects on OIT-Induced Changes

**Figure 5:**
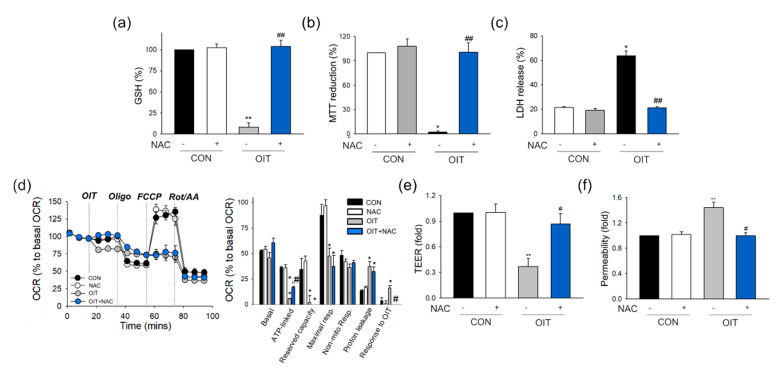
Reversal effects of N-acetyl L-cysteine (NAC) against OIT-mediated effects on GSH level, cell viability, mitochondrial metabolic activity, and endothelial barrier function. (**a**–**c**) bEnd.3 cells were pre-treated with NAC for 2 h, and then, the media were replaced by OIT (25 μM)-containing media. (**a**) The level of reduced GSH was determined in cells 24 h after OIT treatment (*n* = 3). (**b**,**c**) The extent of (**b**) MTT reduction and (**c**) LDH release was measured in cells 24 h after OIT (25 μM) treatment (*n* = 3). (**d**) bEnd.3 cells were pre-treated with NAC for 1 h and incubated for an additional 1 h with assay media containing NAC during degassing. OIT was then acutely injected into the cells. The profile of the OCR was plotted, and parameters for mitochondrial respiration were calculated (*n* = 3–4). (**e**,**f**) Measurement of (**e**) TEER and (**f**) FITC-dextran permeability was conducted in bEnd.3 cells 24 h after OIT (25 μM) treatment with or without NAC pre-treatment. Data are presented as mean ± SEM. * *p* < 0.05, ** *p* < 0.01 vs. CON (control); # *p* < 0.05, ## *p* < 0.01 vs. OIT-treated cells.

N-acetyl L-cysteine (NAC) is a well-established antioxidant that exhibits reducing activity by acting as a precursor of reduced GSH [47]. To examine the role of GSH depletion and mitochondrial dysfunction in OIT treatment, we pre-treated cells with NAC for 2 h, followed by OIT treatment. As shown in Figure 5, NAC pre-treatment significantly reversed the effects of OIT on the level of reduced GSH (Figure 5a), MTT reduction (Figure 5b), and LDH release (Figure 5c). NAC also significantly modified the effects of OIT on mitochondrial oxygen consumption (Figure 5d). The direct decrease in OCR by OIT was significantly reversed by NAC pre-treatment. ATP-linked respiration, which significantly decreased after OIT treatment, was significantly restored by NAC (Figure 5d). Along with the restoration of mitochondrial bioenergetics, OIT-induced dysfunction in the endothelial barrier was significantly reversed by NAC pre-treatment, as measured by TEER (Figure 5e) and FITC-dextran permeability (Figure 5f).

## 3. Discussion

As the personal and industrial use of disinfectants has increased, there have been many safety issues regarding the health effects of biocidal substances [48,49,50,51]. Several studies have reported that IT biocides induce inflammation and allergic effects [2,12,14,52]. The European Chemicals Agency (ECHA) proposed a harmonized classification of OIT, which is a potent antibacterial substance that is corrosive to skin and causes serious eye damage [53]. In addition to these local effects, the United States Environmental Protection Agency (US EPA) has reported that systemic symptoms were observed in animals treated dermally with OIT [54], supporting that OIT may enter the blood circulation after exposure. Although OIT may be absorbed by dermal, oral, and inhalation routes, its effects on systemic organs, including the vascular system, which is a critical target organ for systemically absorbed circulating chemicals, are still unknown.

The BBB, formed by brain endothelial cell (EC) lining, is a barrier that plays a protective role in brain homeostasis by regulating the flux of endogenous circulating substances or xenobiotics between the blood and brain [20,55,56,57,58]. Growing evidence indicates that the functional integrity of the BBB could be affected by blood-circulating substances [21,58,59,60]. Impairment of BBB function can lead to neurological damage, such as neuronal death and synaptic dysfunction, and is associated with various neurodegenerative and neurovascular diseases [61,62,63]. Since the brain ECs show relatively higher ATP demands than other cells, sustaining normal mitochondrial function is crucial for BBB function. Furthermore, accumulating evidence has shown that mitochondria functional impairment is closely related to BBB dysfunction and pathology of neurovascular diseases [64]. Considering that the brain endothelium is continuously exposed to substances entering the systemic circulation, substances that disturb the mitochondria-associated signaling pathway or mitochondrial function may trigger BBB dysfunction and CNS damage [65,66].

Protein SNO is a post-transcriptional modification mediated by pathological NO signaling cascades [34]. Accumulating evidence has shown that aberrantly SNO-modified proteins contribute to neurodegenerative diseases [67,68]. Several studies have shown that SNO modification reduces the activity of enzymes involved in glycolysis and oxidative phosphorylation [69,70,71,72]. However, the exact role of protein SNO modification is still unclear. Molina et al. (1992) reported that the activity of glyceraldehyde-3-phosphate dehydrogenase, an enzyme involved in glycolysis, was decreased by SNO. On the other hand, Chouchani et al. (2013) reported that mitochondrial protein SNO is a cardioprotective mechanism against cardiac ischemia-reperfusion injury, as it decreases the activity of complex I in the ETC. Furthermore, several mitochondria-targeted therapeutic compounds containing S-nitrosothiols have cardioprotective effects [73,74,75]. Therefore, SNO modification of enzymes might not affect the cell uniformly. To understand the effects of SNO on cellular homeostasis, a target-specific approach is needed. In this study, we observed that SNO modification of cellular proteins significantly increased, while the bioenergetic function significantly decreased in OIT-treated brain ECs. These results are consistent with those of previous studies, which showed that increasing levels of SNO-modified proteins were related to a decrease in ATP production. Identifying the target of SNO that contributed to the reported IT biocide-mediated bioenergetic impairments, should be investigated in future studies.

Mitochondria are organelles involved in energy production, cell death, and biosynthetic processes [44]. During a cascade of redox reactions, the ETC generates proton motive force, and this is utilized by ATP synthase (complex V) to produce ATP via oxidative phosphorylation. Mitochondrial uncoupling by FCCP results in a large increase in oxygen consumption in normal conditions, however, both ATP production measured by oligomycin addition and the increases in respiration upon uncoupling can be abolished in cells with mitochondrial depolarization [76]. This might help to understand the OCR pattern affected by OIT (Figure 3d), but the identification of the precise target of OIT in ETC modulation will require further investigation. It is in line with the observation by NAC addition in Figure 5d. While the direct responses of OIT on OCR was significantly reversed by NAC, neither OIT-affected ATP production nor maximal respiration was recovered by NAC, suggesting that further studies are necessary. As oxygen is not only the terminal electron acceptor of the mitochondrial ETC but also a source of ROS, the electron transfer process is expected to be tightly regulated by the balance between oxygen consumption and ATP synthesis. In this study, we observed that OIT-exposed cells showed increased mitochondrial ROS formation and decreased oxidative phosphorylation. ETC dysfunction is known to cause excessive formation of mitochondrial ROS [24,30]. OIT might disturb the electron transfer process during ATP synthesis, which might increase mitochondrial ROS. Interestingly, studies regarding mitochondrial injuries have shown that generation of mitochondrial ROS might lead to the alterations in MMP, but also depolarized MMP propagate mitochondrial ROS generation, which is called ROS-induced ROS release. These crosstalk between inter-mitochondrial networks eventually amplify the loss of mitochondrial function [77,78].

Accumulating evidence has shown that autophagy is an important mechanism for maintaining homeostasis in brain ECs [19]. Autophagy is involved in the adaptive or defensive response against stress conditions, including starvation or oxidative stress [79,80]. However, impairment of autophagic flux has been observed under pathologic conditions, including neurodegenerative diseases [81,82]. In our previous studies [20,21], we demonstrated that autophagy activated by hypoxia or methylglyoxal contributes to endothelial dysfunction by degrading tight junction proteins or mitochondria. Interestingly, dysregulated autophagy, either excessive or compromised, is known to be one of the major mechanisms of xenobiotic toxicity (He et al., 2020). In this study, we elucidated whether OIT, which impaired the function of mitochondria, could also contribute to endothelial dysfunction by activating autophagy. We observed that the formation of autophagosome increased and co-localized with mitochondria in OIT-treated bEnd.3 cells. We also demonstrated that autophagy could be activated by biocide-induced stress, including mitochondrial ROS and mitochondrial damage. Increased proton leakage and mitochondrial ROS after OIT exposure could trigger mitophagy activation.

Since OIT significantly decreased the levels of reduced GSH and bioenergetic parameters of mitochondrial ETC, we attempted to reverse these OIT-induced effects with NAC. NAC pre-treatment significantly reversed the effects of OIT on the level of reduced GSH, cell viability, and endothelial permeability. Although some of the OIT-induced changes in bioenergetic parameters, such as ATP-linked OCR, were significantly restored, other parameters such as maximal respiration were not restored by NAC pre-treatment, suggesting that OIT-induced disruption in each step of ETC is mediated by different mechanisms. Further studies are warranted to investigate the specific effects of OIT on each complex in ETC. Nevertheless, NAC significantly restored OIT-induced disruption in cell viability and endothelial barrier function. These results indicate that the decrease in the level of reduced GSH and initial defects of mitochondrial ETC cause OIT-induced endothelial dysfunction.

Here, we have focused on the effects of OIT on mitochondrial injury and TJ proteins to understand impaired permeability in brain ECs. Considering several emerging targets, which play crucial roles in maintaining BBB properties and barrier function, further studies warrant the alteration of these targets by OIT or IT biocides. These emerging targets can include adherens junctions (AJ) such as vascular endothelium cadherin (VE-cadherin). It is generally accepted that TJ seals the inter-endothelial cleft, while the AJ are essential for initiating and maintaining endothelial cell–cell contacts and promoting their maturation before TJ formation [83,84]. VE-cadherin is known to be critical in neuroinflammation and BBB dysregulation [83], and it would be interesting to elucidate the effects of IT biocide on VE-cadherin. Another target may include transcriptionally regulated molecules such as vascular endothelial growth factors (VEGF). The VEGF regulates TJs and endothelial endocytosis and causes a subsequent increase in vessel permeability [85]. The role of VEGF in BBB breakdown has been mainly studied in pathological conditions such as neuroinflammation and ischemic stroke [86,87]. It could be expanded to studies on xenobiotic-induced BBB damage for a better understanding of xenobiotic-associated neurovascular diseases.

There are several limitations in this study. The dynamics of cell damage and dysfunction are complicated, and the hallmarks manifested by specific alterations in cell injuries, i.e., mitochondrial enzyme activity as observed by MTT or cell membrane perturbation as found in LDH assay, are not in the same line of evidence for dying or dead cells. The treatment of 25 μM OIT induced cell death (Figure 1c,d), and the decreased cell viability itself may contribute to the increased permeability (Figure 1e,f), not critically mediated by mitochondrial damage and TJ degradation. Reversal effects of NAC against OIT-induced functional damages (Figure 5e,f) may be mediated, at least in part, by the reversed cytotoxicity (Figure 5b,c). We would like to point out that the mitochondrial metabolic damage may precede the membrane perturbation and barrier dysfunction, based on the time sequence and the extent of the results from OIT-induced changes in MTT and the LDH assay (Figure 1c,d).

To conclude, we investigated the effects of OIT, which is a commonly used biocide, on brain ECs. OIT decreased brain EC viability and induced barrier integrity impairment and tight junction degradation in brain ECs. Cellular thiol modification, mitochondrial damage, and excessive activation of mitophagy are crucial mechanisms of OIT-induced endothelial dysfunction. We believe that the observations in this study contribute to understanding the mechanisms of OIT-associated health effects and suggest that consistent exposure to this compound may pose a risk of vascular or neurological diseases.

## 4. Materials and Methods

### 4.1. Materials

OIT was purchased from Tokyo Chemical Industry (Tokyo, Japan). Thiazolyl blue tetrazolium bromide (MTT), fluorescein isothiocyanate (FITC)-dextran, and normal donkey serum (NDS) were purchased from Sigma Aldrich (St. Louis, MO, USA). Fluorescent indicators of JC-1, MitoTracker Red CMXRos, and MitoSOX Red mitochondrial superoxide indicator were purchased from Invitrogen (Burlington, ON, Canada). Fluorescein isothiocyanate (FITC)-labeled annexin V (annexin V-FITC) was purchased from BD Pharmingen (San Diego, CA, USA).

### 4.2. Cell Culture

bEnd.3 cells (ATCC, Manassas, VA, USA) from the mouse brain endothelial cell line were used for in vitro BBB experimental models. The cells were maintained in Dulbecco’s modified Eagle’s medium (Welgene, Daegu, Korea) with 10% fetal bovine serum (FBS; Mediatech Inc., Manassas, VA, USA) and 1% penicillin/streptomycin (Welgene). Cells were maintained in a humidified incubator at 37 °C and 5% CO_2_. To confirm the in vitro BBB characters, we used cells with a high confluence (>90%) by microscopic observation. We monitored the TEER values, which are comparable to those previously published with immortalized or primary brain ECs [88].

### 4.3. Measurement of Cell Viability with MTT Reduction and LDH Assay

bEnd.3 cells were seeded at a density of 0.5 × 10^4^ cells per well in 96-well plates. To measure the release of lactate dehydrogenase (LDH) after OIT treatment, we used a CytoTox 96^®^ assay kit (Promega, Madison, WI, USA) following the manufacturer’s instructions. At the end of OIT treatment, the supernatant was collected, and LDH substrates were added after centrifugation. Cells treated with the lysis solution were used as a positive control. After 30 min of incubation, the reaction was terminated by adding stop solution, and the absorbance was determined using an EnSpire multimode spectrophotometer (PerkinElmer, Santa Clara, CA, USA) at 490 nm. After the supernatant was collected, the MTT reduction assay was performed [35]. The cells were treated with 0.5 mg/mL MTT and incubated for 2 h. After dissolving the formazan in dimethyl sulfoxide, the absorbance at 570 nm was measured using a spectrophotometer (PerkinElmer).

### 4.4. In Vitro Permeability Assay

An in vitro permeability assay was performed as previously described [21]. A 0.4 µm pore polycarbonate membrane insert (Corning, New York, NY, USA) was used for the assay, and cells were seeded at a density of 0.2 × 10^5^ cells per well and maintained for 6 d. Trans-endothelial electrical resistance (TEER) and FITC-dextran (m.w. = 4000 Da, Sigma Aldrich) permeability were examined at the end of OIT treatment (24 h). After adding 20 µg/mL FITC-dextran to the apical side of the insert for 30 min, the fluorescence of FITC-dextran on the basolateral side was measured with an EnSpire multimode spectrophotometer (PerkinElmer) using excitation and emission wavelengths of 490 and 520 nm, respectively. An EVOM2 voltohmmeter (World Precision Instruments, Sarasota, FL, USA) was used to measure TEER, and the blank value was subtracted from the data.

### 4.5. Immunofluorescence Staining

Cells were seeded on 8-well chambered cover glass (Thermo Fisher Scientific, Rochester, NY, USA) at a density of 0.9 × 10^4^ cells per well. Ice-cold methanol and acetone were used for fixation and permeabilization, respectively. The sample was blocked with 5% NDS-containing phosphate-buffered saline for 1 h at room temperature. After blocking, the sample was incubated overnight with primary antibodies diluted in 1% NDS in PBS at 4 °C. Fluorescent secondary antibodies diluted in 1% BSA were used for visualization. A K1-Fluo confocal microscope (Nanoscope Systems, Daejeon, Korea) was used to detect fluorescence, and the colocalization of the signal was analyzed using the Imag J plugin JACoP.

To detect the mitochondrial membrane potential (MMP), we used the JC-1 dye (Invitrogen). bEnd.3 cells were seeded on the chambered cover glass and incubated until confluent, as described above. After OIT treatment, the cells were treated with 10 μM JC-1 and incubated at 37 °C for 20 min. Fluorescence was recorded using a K1-Fluo confocal microscope (Nanoscope Systems) immediately after staining.

### 4.6. Total Cellular and Mitochondrial ROS Detection Assays

To detect the total cellular ROS, we used the 2′, 7′7′-dichlorofluorescein diacetate (DCF-DA) cellular ROS detection assay kit (Abcam, Cambridge, MA, USA), as previously described (Min et al., 2020). Cells were seeded on 96-well plates at the same density as described above and incubated to confluence. DCF-DA (25 μM) was added to the cells 30 min before OIT treatment. After OIT treatment, the fluorescence was read at 485/535 nm.

To measure mitochondrial ROS formation, we used the MitoSOX Red mitochondrial superoxide indicator (Invitrogen). bEnd.3 cells were seeded on the chambered cover glass and incubated until confluent, as described above. After OIT treatment, the cells were treated with 5 μM of MitoSOX and incubated at 37 °C for 10 min. The cells were counterstained with 0.15 μg/mL 4,6-diamidino-2-phenylindole (DAPI; Vector Laboratories, Burlingame, CA, USA) after fixation and permeabilization with 2% paraformaldehyde and ice-cold acetone, respectively. Fluorescence was detected using a K1-Fluo confocal microscope (Nanoscope Systems) and Image J was used for quantification.

### 4.7. GSH Assay

To measure the levels of the reduced form of GSH, we used the GSH-Glo Glutathione Assay kit (Promega) following the manufacturer’s instructions. Briefly, the cells were seeded on 96-well plates at the same density as described above. After OIT treatment, cells were treated with the reaction buffer containing glutathione S-transferase and Luciferin-NT for 30 min. After incubation, luciferin detection reagent was added, and luminescence was measured after 15 min using an EnSpire multimode spectrophotometer (PerkinElmer).

### 4.8. Cytosol/Mitochondria Fractionation

Mitochondrial fractionation was performed as previously described [21]. Briefly, the cells were collected after trypsinization and washed once with PBS. Collected cells were lysed in extraction buffer containing protease. After incubating for 10 min on ice, lysates were centrifuged at 700× *g* for 10 min at 4 °C. Collected supernatants were centrifuged at 10,000× *g* for 30 min at 4 °C, and the cytosolic fraction (supernatants) was collected. The mitochondrial fraction (pellets) was resuspended in the extraction buffer, and the protein amount of each fraction was measured by a bicinchoninic acid protein assay (BCA Protein Assay Kit; Thermo Fisher Scientific, Rockford, IL, USA).

### 4.9. Measurement of Bioenergetic Function

The bioenergetic function in bEnd.3 cells was measured after acute injection of OIT using the XFp analyzer and Mitostress test kit (Agilent, Santa Clara, CA, USA). Changes in the extracellular acidification rate (ECAR) and oxygen consumption rate (OCR) were simultaneously monitored by the sequential injection of OIT and modulators of the mitochondrial ETC. Briefly, bEnd.3 cells were seeded on an XFp miniplate (Agilent) at a density of 1.6 × 10^5^ cells/well. After confluence, the cells were incubated with an assay medium (DMEM at pH 7.4; Agilent) containing 5.5 mM glucose, 2 mM L-glutamine, and 1 mM sodium pyruvate. Before analysis, the cells were incubated for 1 h in a non-CO2 incubator for degassing. Next, 2.5 μM oligomycin, 2.0 μM carbonyl cyanide-4-(trifluoromethoxy)phenylhydrazone (FCCP), and 1.0 μM rotenone/antimycin A (Rot/AA) were used as ETC modulators and sequentially injected after OIT treatment. Bioenergetic parameters were calculated from the OCR profile as previously described [21,89].

### 4.10. Analysis of Mitochondrial Mass

To measure the mitochondrial mass, nonyl acridine orange (NAO; Molecular Probes, Eugene, OR, USA), a fluorescent probe that accumulates in the mitochondria, was used [90]. OIT-exposed cells were stained with 1 μM NAO and incubated for 15 min. After incubation, the fluorescence was read with an EnSpire multimode spectrophotometer (PerkinElmer) at 490/520 nm. The data were normalized by the protein amount in each well.

### 4.11. Western Blot

Western blot analysis was performed as previously described [20,21]. Briefly, OIT-exposed cells were lysed in RIPA buffer containing protease and a phosphatase inhibitor cocktail. The protein samples were quantified using a BCA protein assay and mixed with Laemmli buffer containing 5% 2-mercaptoethanol. Sodium dodecyl sulfate-polyacrylamide gel electrophoresis (SDS-PAGE) was used to separate the proteins and was then transferred to the PVDF membrane. The membrane was blocked with 5% bovine serum albumin (BSA) and attached with a primary antibody against claudin-5, ZO-1, voltage-dependent anion channel (VDAC), and LC3B and incubated at 4 °C overnight. After washing three times, HRP-conjugated secondary antibodies were attached to the corresponding target species. Protein bands were detected after adding SuperSignal West Pico/Femto chemiluminescent substrate (Thermo Fischer Scientific).

### 4.12. Protein SNO Analysis

To measure the SNO of proteins, we used PierceTM S-nitrosylation Western Blot Kit (Thermo Fisher Scientific) following the manufacturer’s instructions. The cells were seeded on 6-well plates at a density of 0.9 × 10^4^ cells per well and incubated until confluent. After OIT treatment, the cells were lysed with HENS buffer and sonicated briefly. The protein amount was measured in the supernatant after centrifugation at 10,000× *g* for 10 min. To block free cysteine thiols, 20 mM of S-methyl methanethiosulfonate (MMTS) was added. After 30 min incubation, the protein was precipitated by adding ice-cold acetone to remove residual MMTS. The precipitated proteins were resuspended in HENS buffer and incubated with 0.4 mM iodoTMT (Tandem Mass TagTM) reagent and 40 mM of sodium ascorbate for 1 h. After incubation, the Laemmli sample buffer was added, and the eluted sample was heated for 5 min at 95 °C. Samples were then analyzed by western blotting against anti-TMT.

### 4.13. Flow Cytometry

bEnd.3 cells were seeded on 6-well plates at the same density as described above. After OIT treatment, cells were trypsinized and gently washed twice for further analysis. Harvested cells were stained with 10 μM JC-1 or 10 μg/mL PI/annexin V-FITC (5%, *v*/*v*) fluorescent dye and incubated at 37 °C. Fluorescence was detected using a Guava EasyCyte 8 flow cytometer (Luminex).

For the caspase-3 activity assay, FITC-DEVD-FMK (Biovision, Milpitas, CA, USA), the fluorescent chemicals irreversibly bound to activated caspase-3 in apoptotic cells, was used. Harvested cells were treated with 2 μM FITC-DEVD-FMK and incubated for 30 min at 37 °C. Fluorescence was detected using a Guava EasyCyte 8 flow cytometer after washing twice.

### 4.14. Transmission Electron Microscopy (TEM)

bEnd.3 cells were seeded on 6-well plates at the same density as described above. Cells were fixed with 2% glutaraldehyde at 4 °C after trypsinization and washed twice. Fixed cells were post-fixed with 2% osmium tetroxide after washing and serially dehydrated with 40, 65, 90, and 100% ethanol. Propylene oxide (PO) was used for transition, and infiltration was performed serially with PO and Spurr’s resin 1:1, 1:2 solution, and only with Spurr’s resin. Samples were observed with JEM1010 TEM (JEOL) after overnight incubation at 70 °C for polymerization.

### 4.15. Statistical Analysis

All data are expressed as the standard error of the mean (SEM). To test the statistical significance between groups, a Student’s t-test, one-way analysis of variance (ANOVA), and Tukey’s post hoc test were performed. SPSS version 24 (SPSS Inc., Chicago, IL, USA) was used for statistical analysis, and a *p*-value < 0.05 was regarded as statistically significant in all analyses.

## Data Availability

Not applicable.

## Abbreviations:

AJ	Adherens junctions
BBB	Blood–brain barrier
BCA	Bicinchoninic acid
CNS	Central nervous system
DAPI	4,6-Diamidino-2-phenylindole
DCF-DA	Dichlorofluorescein diacetate
EC	Endothelial cell
ECHA	European Chemicals Agency
ETC	Electron transport chain
FCCP	Carbonyl cyanide-4-(trifluoromethoxy) phenylhydrazone
FITC	Fluorescein isothiocyanate
GSH	Glutathione
IT	Isothiazolinone
LDH	Lactate dehydrogenase
MMP	Mitochondrial membrane potential
MMTS	S-methyl methanethiosulfonate
MTT	Thiazolyl blue tetrazolium bromide
NAC	N-acetyl L-cysteine
NAO	Nonyl acridine orange
NO	Nitric oxide
OCR	Oxygen consumption rate
OIT	2-N-octyl-4-isothiazolin-3-one
PS	Phosphatidylserine
ROS	Reactive oxygen species
Rot/AA	Rotenone/antimycin A
SNO	S-nitrosylation
TEER	Trans-endothelial electrical resistance
TEM	Transmission electron microscopy
TJ	Tight junction
US EPA	United States Environmental Protection Agency
VDAC	Voltage-dependent anion channel
VEGF	Vascular endothelial growth factors
VE-cadherin	Vascular endothelium cadherin
ZO-1	Zonula occludens-1
